# Diabetes-associated Alterations in the Gut Microbiota–metabolite Profile of NAFLD Under Complex Clinical Conditions^☆^

**DOI:** 10.1016/j.jceh.2026.103637

**Published:** 2026-08-19

**Authors:** Jieying Zeng, Keen Yang, Huaiyu Wu, Ziwei Lin, Lisi Liao, Fajin Dong, Yaxin Zhu

**Affiliations:** ∗ Department of Ultrasound, Shenzhen People's Hospital (The Second Clinical Medical College, Jinan University, The First Affiliated Hospital, Southern University of Science and Technology), Shenzhen, Guangdong, 518020, China; † Department of Endocrinology, Shenzhen People's Hospital (The Second Clinical Medical College, Jinan University, The First Affiliated Hospital, Southern University of Science and Technology), Shenzhen, Guangdong, 518020, China

**Keywords:** non-alcoholic fatty liver disease, overweight, diabetes, metabolic diseases, gut microbiota

## Abstract

**Background:**

Non-alcoholic fatty liver disease (NAFLD) frequently coexists with obesity and type 2 diabetes (T2D), and the presence of these metabolic conditions contributes to substantial heterogeneity in disease progression and metabolic dysfunction. Gut microbiota and their derived metabolites are increasingly recognized as important mediators linking NAFLD with systemic metabolic disorders; however, how overweight and T2D differentially shape gut microbiota–metabolite alterations in NAFLD under complex clinical conditions remains unclear.

**Methods:**

In this study, 66 patients with NAFLD (including 27 with T2D) and 26 healthy controls were enrolled. All participants underwent liver ultrasound–based assessment, biochemical testing, and fecal sample collection on the same day. Gut microbiota composition was analyzed using 16S ribosomal RNA gene sequencing, and fecal metabolites, including short-chain fatty acids and bile acids, were quantified using light chromatography–tandem mass spectrometry. Participants were stratified according to overweight and diabetes status to compare microbiota and metabolite profiles across metabolic subgroups.

**Results:**

Distinct alterations in gut microbiota and metabolites were observed among NAFLD patients with different metabolic conditions. Patients with T2D showed a butyrate-related pattern in exploratory analysis, including nominally lower butyrate and isobutyrate levels and lower abundance of several butyrate-producing genera, particularly *Lachnospira*, *Roseburia*, and *Anaerostipes*, compared with non-diabetic NAFLD patients. *Faecalibacterium* was lower in NAFLD patients than in healthy controls but did not show a clear diabetes-specific decrease. Bile acid–related metabolites and associated bacterial taxa also showed exploratory patterns in diabetic NAFLD patients. Overall, diabetes was associated with more evident gut microbiota differences and exploratory metabolite patterns than overweight alone within the NAFLD population.

**Conclusion:**

Under complex clinical conditions, overweight and T2D were associated with different gut microbiota profiles and exploratory metabolite patterns in patients with NAFLD, with diabetes showing a stronger association than overweight alone. These exploratory findings highlight the importance of considering metabolic comorbidities when interpreting gut microbiota–metabolite changes in imaging-defined NAFLD and provide preliminary insight into microbial–metabolic patterns underlying disease heterogeneity.

Non-alcoholic fatty liver disease (NAFLD) is defined by excessive hepatic fat accumulation in the absence of secondary causes, such as significant alcohol intake, viral hepatitis, drug-induced liver injury, or autoimmune liver disease. In clinical practice, NAFLD is commonly diagnosed using imaging-based methods.^1^^,^^2^ The global prevalence of NAFLD is approximately 25%–30%^3^ and continues to increase.^4^ Its associated mortality rate has surpassed that of viral hepatitis, posing a serious challenge to healthcare systems in various countries.^5^

NAFLD is not only the main cause of liver-related diseases, but also an important contributor to various extra-hepatic chronic diseases, such as cardiovascular diseases, type 2 diabetes (T2D), and chronic kidney diseases.^6^ Obesity was previously considered the main cause of NAFLD. However, with deeper research into NAFLD, it has been found that about 30% of non-obese patients (judged by body mass index [BMI]) also have NAFLD, and their pathological changes are consistent.^7^ Dyslipidemia is common in NAFLD, but hepatic steatosis does not always parallel circulating lipid levels. In clinical practice, lipid-lowering therapy is considered when dyslipidemia is present, whereas lifestyle modification remains the cornerstone of management.^8^ These observations suggest that NAFLD is metabolically heterogeneous and may be influenced by obesity, age, glycemic status, and lipid metabolism.^7^^,^^8^

With advances in metagenomic technologies, gut microbiota dysbiosis has been increasingly linked to chronic liver diseases and extra-hepatic metabolic disorders through the gut–liver axis.^9^ Gut microbiota has been explored as a potential target in metabolic diseases, but its clinical use remains limited and requires stronger evidence.^10^ Research on developing early diagnostic and prognostic models for diseases based on gut microbiota already exists, but the widespread implementation of these models is hindered because gut microbiota is easily influenced by factors such as region, dietary habits, and medications.^11^

In this context, we aimed to determine whether gut microbiota and metabolite features associated with overweight and diabetes could be consistently identified using imaging-based stratification combined with objective clinical indicators in a complex clinical cohort. Therefore, we conducted a preliminary exploratory study to compare gut microbiota and metabolite patterns in NAFLD patients with overweight and diabetes and to assess whether these features showed preliminary discrimination ability across metabolic subgroups.

## Materials and methods

### Participants

This was a single-center, prospective, cross-sectional observational study. From June 2021 to June 2022, patients who were newly diagnosed with NAFLD at our hospital were included in the study. This research protocol was conducted in accordance with the Declaration of Helsinki and has been approved by the local ethics committee. All patients provided written informed consent before participating in the study.

Inclusion criteria.

Participants were eligible if they met the following criteria:

◆ age >18 yr and ability to move independently;
◆ completion of routine liver ultrasound, functional ultrasound examination, biochemical laboratory tests, and stool sample collection on the same day;
◆ stool samples were stored at −80 °C within 30 min after collection.

Exclusion criteria.

◆ Participants were excluded, if they met any of the following criteria:
◆ viral hepatitis, drug-induced liver disease, autoimmune liver disease, or other known chronic liver diseases;
◆ evidence of significant liver fibrosis or cirrhosis on ultrasound or elastography-based assessment;
◆ substantial body weight change within the previous 6 mo;
◆ use of antibiotics, probiotics/prebiotics, metformin, other antidiabetic medications, immunosuppressants, hormones, or anti-inflammatory drugs within 1 mo before enrollment. Patients classified as having T2D in this study were newly diagnosed at enrollment and had not received any previous antidiabetic treatment; no diabetic patient was withdrawn from antidiabetic therapy for study participation;
◆ significant alcohol intake, defined as ≥30 g/d for men or ≥20 g/d for women;
◆ history of gastrointestinal surgery or planned gastrointestinal surgery;
◆ pregnancy, recent major cardiovascular events such as myocardial infarction or stroke, or confirmed inflammatory bowel disease, including Crohn’s disease or ulcerative colitis.

All participants were asked to maintain their usual diet and lifestyle before stool collection. Long-term dietary intake and physical activity were not quantitatively assessed, so residual confounding from habitual diet or lifestyle could not be fully excluded.

### Liver Stiffness and Fat Content Assessment

Routine abdominal ultrasound examination was performed using a convex array probe with a frequency of 2–5 MHz (Mindray Resona 7A), and liver stiffness evaluation was conducted using transient elastography (FibroScan)^12^ and real-time shear wave elastography (Mindray Resona 7A).^13^ Liver fat content measurement was assessed using the controlled attenuation parameter (CAP) technology.^14^

Hepatic steatosis was quantitatively assessed using the CAP) and classified as follows:

◆ Normal liver fat content: CAP <248 dB/m
◆ Mild fatty liver (FL1): CAP 248–267 dB/m
◆ Moderate-to-severe fatty liver (FL2): CAP ≥268 dB/m

Liver stiffness was quantitatively assessed using transient elastography and real-time shear wave elastography and classified as follows:

◆ No or minimal fibrosis (F0–F1): E < 7.0 kPa
◆ Significant fibrosis (F2): E 7.0–8.9 kPa
◆ Advanced fibrosis (F3): E 9.0–12.4 kPa
◆ Liver cirrhosis (F4): E ≥ 12.5 kPa

Liver biopsy was not performed in this study. Therefore, the FL1 and FL2 groups represent CAP-defined steatosis categories rather than histologically confirmed non-alcoholic steatohepatitis (NASH), inflammatory activity, or fibrosis stages. Liver stiffness measurements were used to screen for significant fibrosis or cirrhosis but were not considered a substitute for histological staging.

### Clinical and Biochemical Parameters

Age, sex, waist circumference (WC), BMI, glucose, glycated hemoglobin (HbA1c), low-density lipoprotein (LDL), triglyceride, high-density lipoprotein (HDL), total cholesterol, γ-glutamyl transpeptidase, alanine aminotransferase, aspartate aminotransferase, alkaline phosphatase, albumin, total protein, direct bilirubin, total bilirubin, white blood cell, and platelet were collected simultaneously. BMI was calculated as weight divided by height squared (kg/m^2^). In this study, overweight was defined as BMI >25 kg/m^2^. This cutoff was chosen according to the conventional international BMI classification to avoid inconsistencies arising from different regional BMI definitions during subgroup analysis. WC was measured horizontally at the approximate midpoint between the lower margin of the last palpable rib and the top of the iliac crest.

### Stool Sample Detection and Database Construction

Stool samples were collected from all participants and stored at −80 °C until further processing. Microbial genomic DNA was extracted from fecal samples using a standardized commercial DNA extraction kit according to the manufacturer’s instructions. The V3–V4 hypervariable regions of the bacterial 16S ribosomal RNA gene were amplified using universal primers, followed by high-throughput sequencing on an Illumina platform.

Raw sequencing reads were filtered to generate clean reads. Low-quality reads were trimmed using a 25-bp sliding window, and reads were removed if the retained length was less than 75% of the original read length. Reads with adapter contamination, ambiguous bases, or low-complexity sequences were also removed. Paired-end reads were merged into tags using FLASH (Fast Length Adjustment of SHort reads) v1.2.11, with a minimum overlap of 15 bp and a maximum mismatch rate of 0.1 in the overlap region. High-quality tags were clustered into operational taxonomic units (OTUs) at 97% sequence similarity using Ultra-fast SEARCH/Ultra-fast PARSE (USEARCH/UPARSE). Chimeric sequences were removed using USEARCH Chimera Detection (UCHIME) against the Gold database, and all tags were mapped back to representative OTU sequences using usearch_global to generate the OTU abundance table. The OTU abundance table was converted to relative abundance for taxonomic composition, differential abundance, and correlation analyses. Alpha diversity rarefaction curves were generated to assess sequencing sufficiency, but the original platform report did not provide a single fixed rarefaction depth.

### Fecal Metabolite Analysis

Fecal metabolite profiling was performed using a liquid chromatography–tandem mass spectrometry platform. Short-chain fatty acids (SCFAs) and bile acids were quantified using stable isotope–labeled internal standards, which were added to each fecal sample prior to extraction. Metabolite concentrations were determined by comparing analyte peak areas with those of the corresponding internal standards, allowing accurate quantification and correction for analytical variability.

All fecal samples were processed using a standardized protocol. Samples were weighed before extraction, and metabolite concentrations were normalized to fecal sample mass. This weight-based normalization minimized variability related to differences in sample amount and supported comparability of metabolite levels across individuals.

### Statistical Method

All statistical analyses were performed using R software (version 3.6.1). Continuous variables are presented as mean ± standard deviation or median (interquartile range), as appropriate. Categorical variables are presented as frequencies (percentages). Differences in baseline clinical characteristics among groups were assessed using one-way analysis of variance with Tukey’s post hoc test for normally distributed continuous variables or the Kruskal–Wallis test with appropriate post hoc comparisons for non-normally distributed continuous variables. Categorical variables were compared using the chi-square test or Fisher’s exact test, as appropriate.

Gut microbial alpha diversity was evaluated using the Simpson index, and between-group differences were assessed using the Kruskal–Wallis test followed by Dunn’’s post hoc test. Beta diversity was calculated using weighted UniFrac distances, and group differences were evaluated using permutational multivariate analysis of variance.

For bacterial taxa and fecal metabolites, overall comparisons among three or more groups were performed using the Kruskal–Wallis test, and pairwise comparisons were performed using the Wilcoxon rank-sum test. For bacterial taxa, differential abundance analyses were performed separately at the phylum and genus levels, and *P* values were adjusted within each taxonomic level using the Benjamini–Hochberg false discovery rate (BH-FDR) method. For fecal metabolites, raw *P* values and BH-FDR–adjusted q values were reported to distinguish nominal findings from statistically supported findings. A q value < 0.05 was considered statistically significant.

Spearman’s rank correlation analysis was used as an exploratory approach to assess associations between gut bacteria, metabolites, and clinical indicators, and correlation heatmaps were generated for visualization. These correlation analyses were not adjusted using multivariable regression models. Random forest analysis was conducted to rank bacterial features by importance, and the top 30 bacterial features were selected for further analysis. Exploratory discrimination models for NAFLD, diabetes, overweight, and combined overweight–diabetes status were constructed using selected bacterial features together with metabolites showing nominal differences. Model performance was summarized using receiver operating characteristic curve (ROC) and area under the receiver operating characteristic curve (AUC) values. No internal validation, such as k-fold cross-validation, bootstrap validation, or repeated random splitting, was performed, and no external validation cohort was available. Therefore, the ROC/AUC values were interpreted only as apparent exploratory performance in the study cohort, not as validated diagnostic performance.

Unless otherwise specified, a two-sided *P*-value <0.05 was considered statistically significant.

## Results

### Baseline Clinical Characteristics of NAFLD Patients and Healthy Controls

A total of 110 participants were initially recruited, including 80 patients with NAFLD and 30 healthy controls. After excluding 14 NAFLD patients because of missing blood biochemistry data or poor fecal sample quality, and 4 controls because of hyperglycemia or hyperlipidemia, 92 participants were included in the final analysis: 66 patients with NAFLD and 26 healthy controls (Supplementary Fig. S1). The NAFLD group comprised 30 patients with mild fatty liver (FL1) and 36 with moderate-to-severe fatty liver (FL2). No evidence of significant fibrosis or cirrhosis was detected based on elastography-based liver stiffness assessment (two functional imaging techniques). Table 1 summarizes the baseline characteristics of patients with NAFLD and healthy controls. Differences in several clinical and biochemical parameters were observed among the study groups.

**Table 1:** Baseline Characteristics and Laboratory Findings of the Participants.

Variables	Total	N	FL1	FL2	*P* (NAFLD)	*P* (DM)	*P* (OB)	*P* (OB-DM)
n	92	26	30	36	–	–	–	–
Age, year	45.82 ± 11.93	46.73 ± 11.14	47.7 ± 13.12	43.58 ± 11.4	–	0.02	–	–
Gender								
Female	42	13	12	17				
Male	50	13	18	19	–	–	–	–
WC, cm	93.25 ± 11.79	83.19 ± 10.52	93 ± 8.24	100.72 ± 9.67	<0.01	<0.01	<0.01	<0.01
BMI, kg/m^2^	24.68 (22.79, 27.35)	21.7 (20.77, 22.63)	24.72 (24.35, 25.59)	27.91 (25.69, 29.81)	<0.01	<0.01	<0.01	<0.01
Glucose, mmol/L	5.34 (4.9, 6.26)	4.97 (4.77, 5.54)	5.36 (4.79, 6.69)	5.62 (5.12, 7.61)	0.01	<0.01	<0.01	<0.01
HbA1c, %	5.5 (5.1, 6.5)	5.1 (4.87, 5.4)	6.2 (5.44, 7.34)	5.52 (5.1, 6.78)	<0.01	<0.01	<0.01	<0.01
LDL, mmol/L	3.03 ± 0.96	3.22 ± 0.59	2.75 ± 1.07	3.11 ± 1.06	–	–	–	–
TG, mmol/L	1.48 (0.92, 2.55)	0.88 (0.76, 1.01)	1.71 (1.26, 3.21)	2.21 (1.7, 4.46)	<0.01	<0.01	<0.01	<0.01
HDL, mmol/L	1.04 (0.89, 1.21)	0.92 (0.88, 1.21)	1.12 (0.98, 1.41)	1.01 (0.88, 1.17)	–	<0.01	<0.01	<0.01
TC, mmol/L	5.16 ± 1.22	4.81 ± 0.86	5.01 ± 1.33	5.53 ± 1.27	–	–	–	–
GGT, U/L	30 (16, 46.5)	15.5 (13.25, 18)	34.5 (25.02, 47.5)	44.5 (32, 65.5)	<0.01	<0.01	0.01	<0.01
ALT, U/L	20 (15, 31)	15.5 (12.5, 18)	20 (15.32, 27.25)	32.5 (21, 58.25)	<0.01	<0.01	<0.01	<0.01
AST, U/L	24.5 (17, 41.7)	18 (14, 30.5)	22 (16.85, 39.5)	35 (25, 46.1)	<0.01	0.02	0.01	<0.01
ALP, U/L	66.15 (58.6, 70.62)	63 (54.25, 67.67)	66.5 (56.25, 68.88)	68.6 (61.75, 79.5)	<0.01	–	<0.01	<0.01
ALB, g/L	43.05 (41.77, 46.4)	42.94 (40.05, 46.25)	43.1 (40.97, 46.2)	43.2 (41.95, 46.55)	–	0.04	–	–
TP, g/L	68.65 (64.17, 73.03)	66.35 (62.02, 72.8)	69.65 (64.47, 71.55)	69.35 (65.58, 73.8)	–	–	–	–
DB, μmol/L	3.2 (2.3, 4.62)	2.25 (1.95, 2.7)	3.58 (2.66, 4.78)	3.75 (3.08, 4.7)	<0.01	<0.01	<0.01	0.04
TB, μmol/L	10.9 (7.95, 14.9)	10.5 (8.2, 12.95)	11.8 (7.55, 14.67)	11.2 (8, 19.45)	–	–	–	–
WBC, x 10^9^/L	6.72 (5.79, 7.96)	6.72 (6.26, 7.97)	6.94 (5.7, 8.28)	6.64 (5.77, 7.81)	–	–	0.03	–
PLT, x 10^9^/L	242 (215, 266.25)	220 (204.75, 240.5)	254 (231, 270.5)	250 (227.25, 270.25)	<0.01	<0.01	0.01	<0.01
FibroScan-CAP, dB/m	260 (212, 291.25)	198.5 (177.25, 210)	255 (248.75, 260)	303.5 (282.5, 320)	<0.01	<0.01	<0.01	<0.01
FibroScan-VCTE, kPa	5.51 ± 1.04	4.85 ± 0.74	5.59 ± 1.06	5.93 ± 0.98	<0.01	<0.01	<0.01	<0.01
STE, kPa	5.6 ± 0.94	5.82 ± 0.87	5.63 ± 0.91	5.41 ± 1	–	–	–	–

N, healthy control group; FL1, mild fatty liver; FL2, moderate-to-severe fatty liver; NAFLD, non-alcoholic fatty liver disease; DM, diabetes mellitus; OB, overweight; OB-DM, overweight with diabetes; WC, waist circumference; BMI, body mass index; HbA1c, glycated hemoglobin; LDL, low-density lipoprotein; TG, triglyceride; HDL, high-density lipoprotein; TC, total cholesterol; GGT, γ-glutamyl transpeptidase; ALT, alanine aminotransferase; AST, aspartate aminotransferase; ALP, alkaline phosphatase; ALB, albumin; TP, total protein; DB, direct bilirubin; TB, total bilirubin; WBC, white blood cell; PLT, platelet; CAP, controlled attenuation parameter; VCTE, vibration-controlled transient elastography; STE, shear wave elastography.

**Note:** Data are shown as mean ± standard deviation (SD), median (interquartile range [IQR]), or n, as appropriate. The second row shows the number of participants in each group. The four *P*-value columns summarize comparisons by CAP-defined steatosis severity, diabetes status, overweight status, and combined overweight–diabetes status. Continuous variables were compared using analysis of variance (ANOVA) or the Kruskal–Wallis test, and categorical variables using the chi-square test or Fisher’s exact test. “—” indicates *P* ≥ 0.05 or not applicable.

### Gut Microbial Diversity Differs Across NAFLD-Related Metabolic Subgroups

After quality control and OTU clustering at a 97% similarity level, 1057 OTUs were identified from 92 samples. A total of 10 bacterial phyla and 40 genera with log10 relative abundance >0.0005 were retained for downstream analyses.

Gut microbial diversity was compared across multiple NAFLD-related metabolic stratifications, including: (a) healthy controls (N, n = 26) vs. mild fatty liver (FL1, n = 30) vs. moderate-to-severe fatty liver (FL2, n = 36); (b) healthy controls (N, n = 26) vs. fatty liver with diabetes (FL-DM, n = 27) vs. fatty liver without diabetes (FL-NDM, n = 39); (c) healthy controls (N, n = 26) vs. fatty liver with overweight (FL-OB, n = 38) vs. fatty liver without overweight (FL-NOB, n = 28); and (d) healthy controls (N, n = 26) vs. fatty liver with overweight and diabetes (FL-OB-DM, n = 16), fatty liver with overweight and without diabetes (FL-OB-NDM, n = 22), fatty liver with non-overweight and diabetes (FL-NOB-DM, n = 11), and fatty liver with non-overweight and without diabetes (FL-NOB-NDM, n = 17).

**Figure 1:**
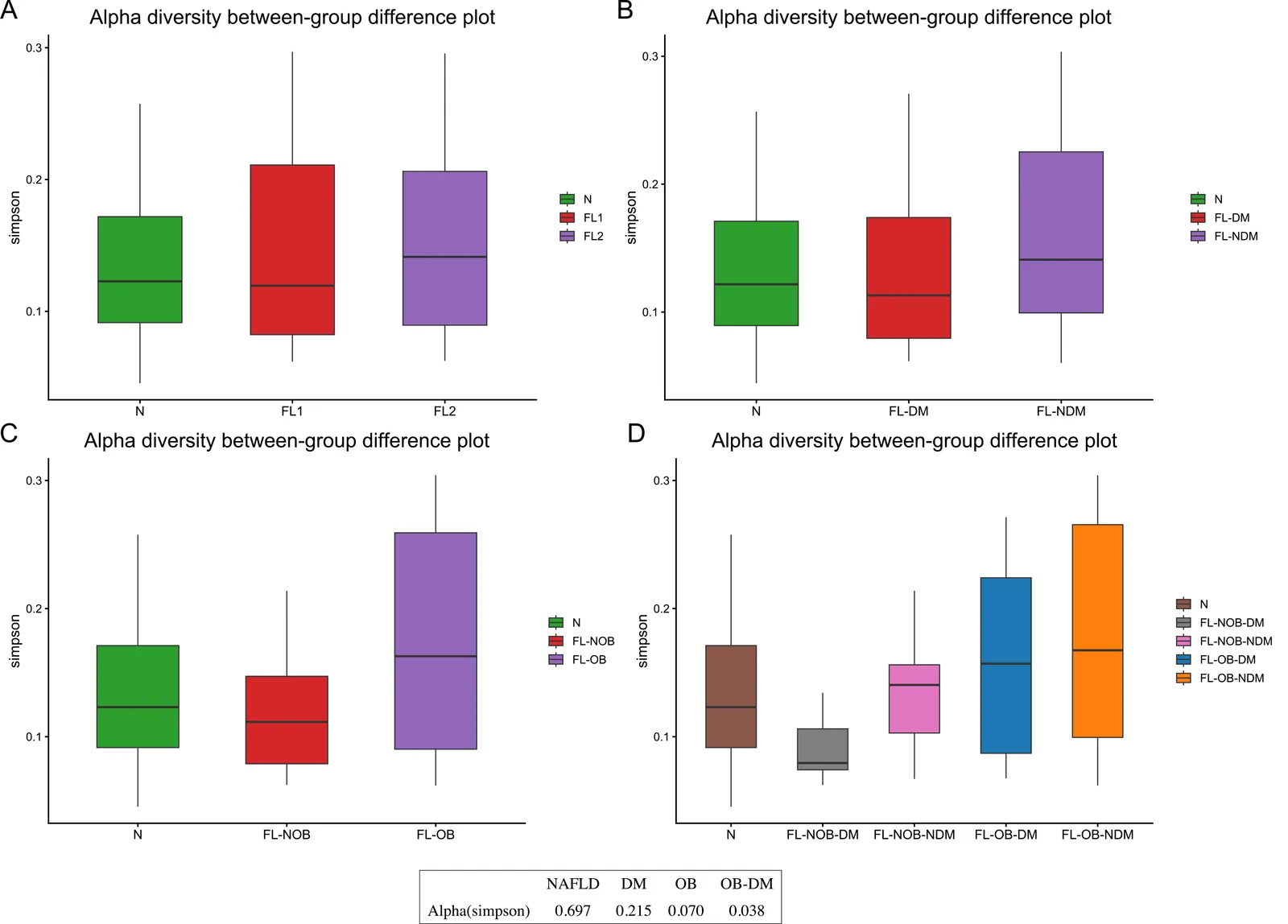
**Alpha diversity results**. A, NAFLD; B, DM; C, OB; D, OB-DM. Alpha diversity was assessed using the Simpson index. Group differences were tested using the Kruskal–Wallis test followed by Dunn’s post hoc test. Sample sizes: N = 26, FL1 = 30, FL2 = 36; FL-DM = 27, FL-NDM = 39; FL-OB = 38, FL-NOB = 28; FL-OB-DM = 16, FL-OB-NDM = 22, FL-NOB-DM = 11, FL-NOB-NDM = 17. NAFLD: Non-alcoholic fatty liver disease; DM: diabetes; OB: overweight; OB-DM: overweight with diabetes; N: healthy control group; FL1: mild fatty liver; FL2: moderate-to-severe fatty liver; FL-DM: fatty liver with diabetes; FL-NDM: fatty liver without diabetes; FL-OB: fatty liver with overweight; FL-NOB: fatty liver without overweight; FL-OB-DM: fatty liver with overweight with diabetes; FL-OB-NDM: fatty liver with overweight without diabetes; FL-NOB-DM: fatty liver with non-overweight with diabetes; FL-NOB-NDM: fatty liver with non-overweight without diabetes.

**Figure 2:**
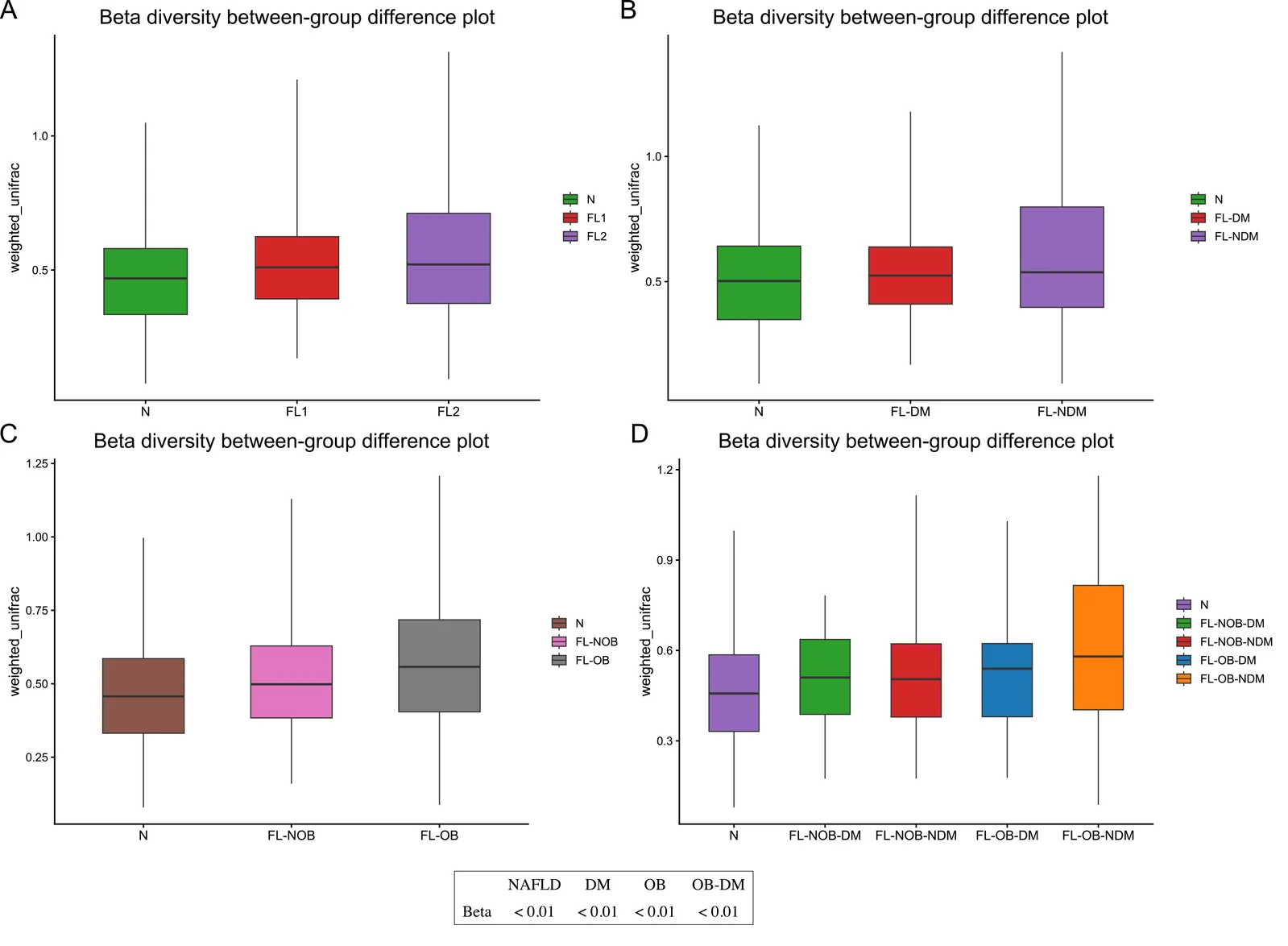
**Beta diversity results**. A, NAFLD; B, DM; C, OB; D, OB-DM. Beta diversity was calculated using weighted UniFrac distances, and group differences were tested using PERMANOVA. Sample sizes are the same as in Figure 1. NAFLD: Non-alcoholic fatty liver disease; DM: diabetes; OB: overweight; OB-DM: overweight with diabetes; PERMANOVA, permutational multivariate analysis of variance; N: healthy control group; FL1: mild fatty liver; FL2: moderate-to-severe fatty liver; FL-DM: fatty liver with diabetes; FL-NDM: fatty liver without diabetes; FL-OB: fatty liver with overweight; FL-NOB: fatty liver without overweight; FL-OB-DM: fatty liver with overweight with diabetes; FL-OB-NDM: fatty liver with overweight without diabetes; FL-NOB-DM: fatty liver with non-overweight with diabetes; FL-NOB-NDM: fatty liver with non-overweight without diabetes.

Alpha diversity was assessed using the Simpson index, and beta diversity was evaluated based on weighted UniFrac distances. Alpha diversity showed no significant overall difference in the CAP-defined steatosis or diabetes-based comparisons. The overweight-based comparison showed a borderline overall difference (*P* = 0.070), whereas the combined overweight–diabetes stratification showed an overall difference (*P* = 0.038; Figure 1). In contrast, beta diversity analysis revealed significant between-group separation across all four stratification schemes (Figure 2).

### Gut Microbiota and Metabolite Alterations Across CAP-Defined Hepatic Steatosis Groups

**Figure 3:**
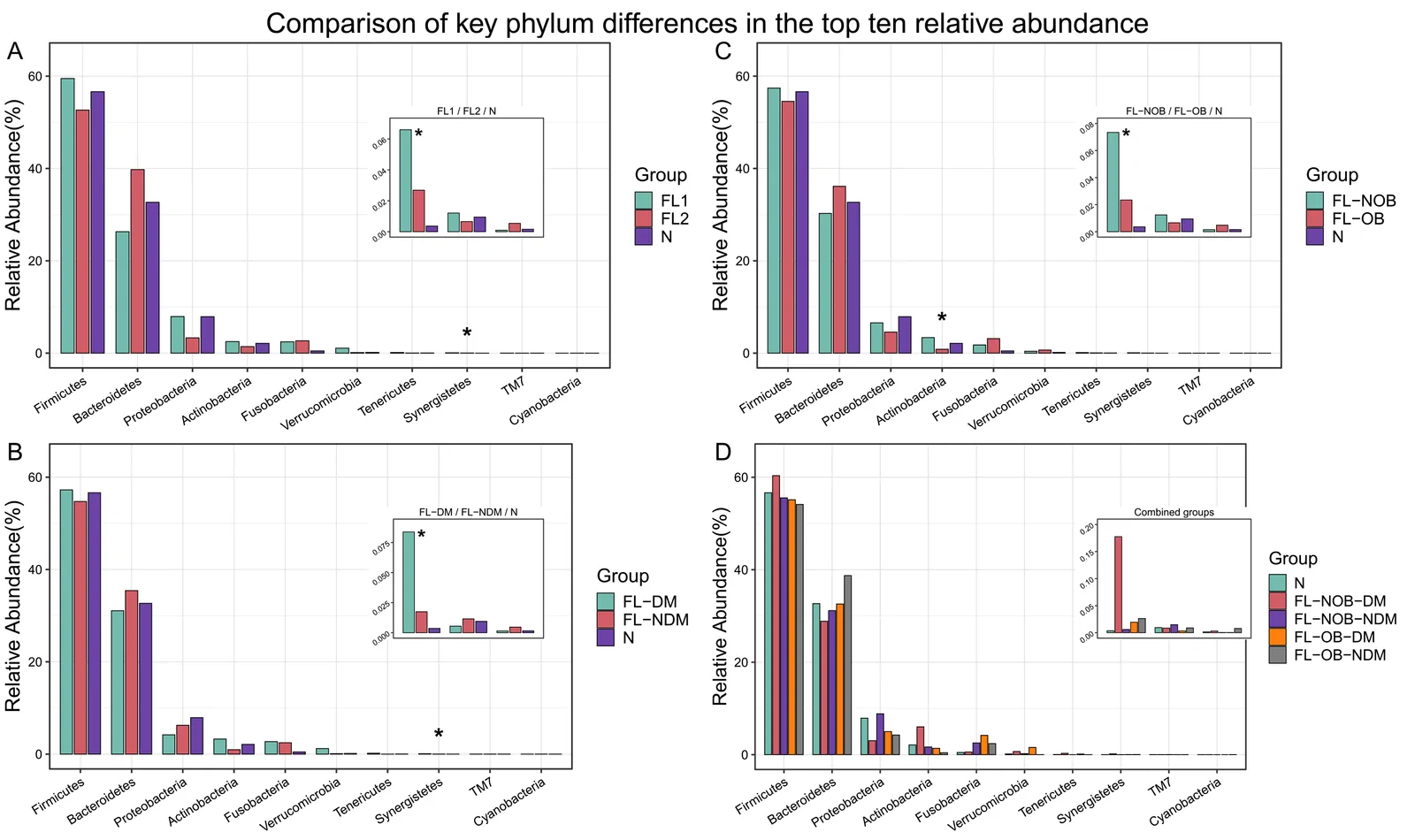
**Comparison of key phylum differences in the top ten relative abundance**. A, NAFLD; B, DM; C, OB; D, OB-DM. The horizontal axis represents the top 10 most abundant bacteria, and the vertical axis represents the relative abundance percentage. An asterisk (∗) indicates a nominal difference in bacterial abundance based on raw *P* < 0.05. Overall group differences were assessed using the Kruskal–Wallis test, and pairwise comparisons were assessed using the Wilcoxon rank-sum test, as appropriate. Sample sizes are the same as in Figure 1. NAFLD: Non-alcoholic fatty liver disease; DM: diabetes; OB: overweight; OB-DM: overweight with diabetes; N: healthy control group; FL1: mild fatty liver; FL2: moderate-to-severe fatty liver; FL-DM: fatty liver with diabetes; FL-NDM: fatty liver without diabetes; FL-OB: fatty liver with overweight; FL-NOB: fatty liver without overweight; FL-OB-DM: fatty liver with overweight with diabetes; FL-OB-NDM: fatty liver with overweight without diabetes; FL-NOB-DM: fatty liver with non-overweight with diabetes; FL-NOB-NDM: fatty liver with non-overweight without diabetes.

**Figure 4:**
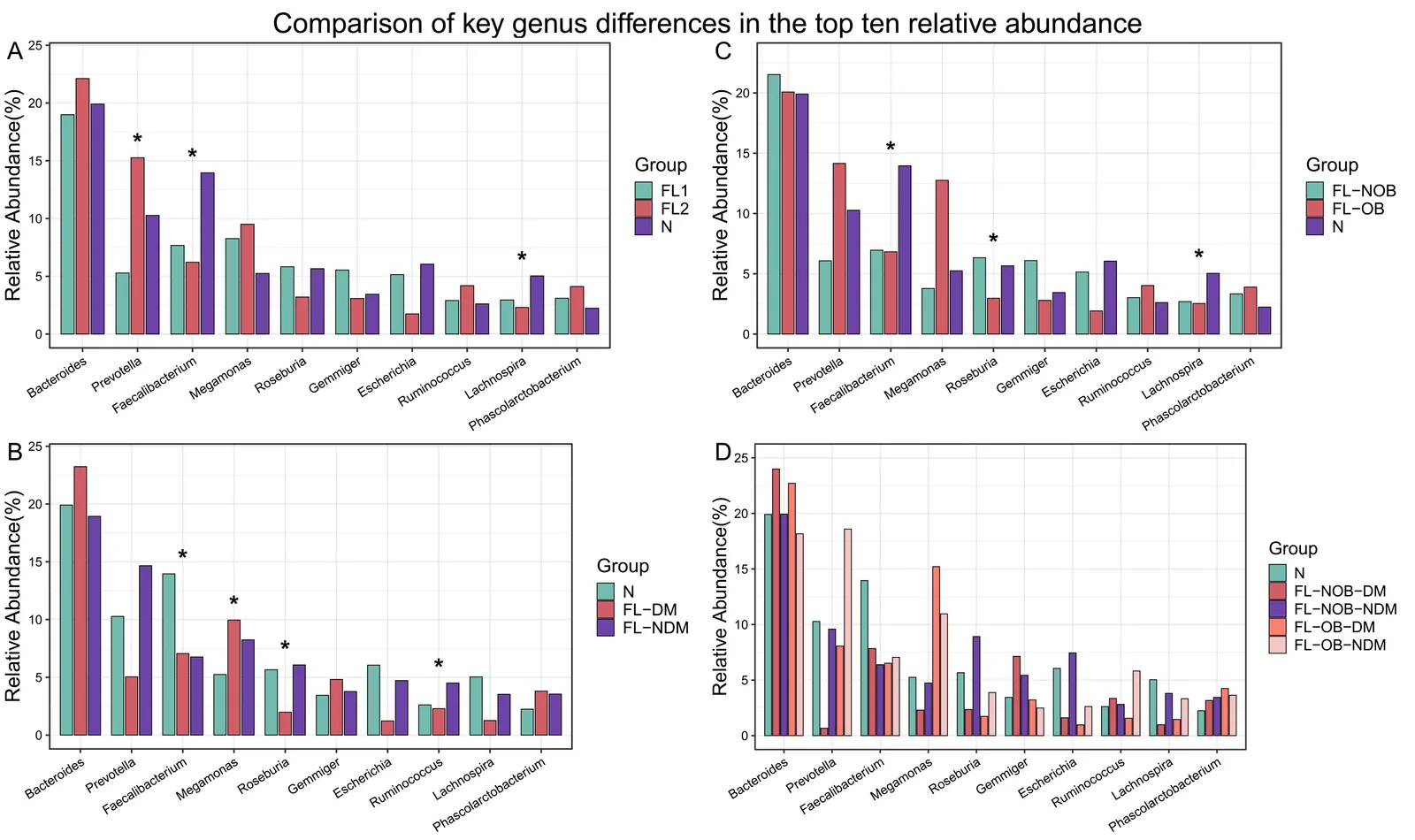
**Comparison of key genus differences in the top ten relative abundance**. A, NAFLD; B, DM; C, OB; D, OB-DM. The horizontal axis represents the top 10 most abundant bacteria, and the vertical axis represents the relative abundance percentage. An asterisk (∗) indicates a nominal difference in bacterial abundance based on raw *P* < 0.05. Overall group differences were assessed using the Kruskal–Wallis test, and pairwise comparisons were assessed using the Wilcoxon rank-sum test, as appropriate. Sample sizes are the same as in Figure 1. NAFLD: Non-alcoholic fatty liver disease; DM: diabetes; OB: overweight; OB-DM: overweight with diabetes; N: healthy control group; FL1: mild fatty liver; FL2: moderate-to-severe fatty liver; FL-DM: fatty liver with diabetes; FL-NDM: fatty liver without diabetes; FL-OB: fatty liver with overweight; FL-NOB: fatty liver without overweight; FL-OB-DM: fatty liver with overweight with diabetes; FL-OB-NDM: fatty liver with overweight without diabetes; FL-NOB-DM: fatty liver with non-overweight with diabetes; FL-NOB-NDM: fatty liver with non-overweight without diabetes.

At the phylum level, Synergistetes abundance was higher in patients with NAFLD than in healthy controls, but no consistent severity-dependent gradient was observed between the FL1 and FL2 groups (Figure 3). At the genus level, eight genera differed among healthy controls and CAP-defined steatosis groups (Table 2). Reductions in several butyrate-producing genera, including *Faecalibacterium*, *Anaerostipes*, and *Lachnospira*, were observed in NAFLD patients compared with healthy controls (Figure 4).

**Table 2:** Differentially Abundant Bacterial Taxa and Exploratory Metabolite Differences Across CAP-Defined Hepatic Steatosis Groups.

	N	FL1	FL2	*P*/FDR _(N vs FL1 vs FL2)_	*P*/FDR _(N vs FL1)_	*P*/FDR _(N vs FL2)_	*P*/FDR _(FL1 vs FL2)_
n	26	30	36				
Phylum							
Synergistetes^a^	0.004	0.066	0.027	0.008/0.110	0.013/0.03	0.002/0.007	–
Bacteroidetes	32.674	26.319	39.768	–	–	–	0.034/0.443
Genus							
*Anaerostipes*	0.221	0.051	0.140	0.001/0.079	∗∗∗/0.026	0.010/0.016	–
*Pseudoramibacter_Eubacterium*	0.000	0.008	0.002	0.001/0.079	∗∗∗/0.002	0.046/0.046	0.040/0.046
*Faecalibacterium*^a^	13.922	7.653	6.203	0.008/0.223	0.028/0.042	0.002/0.006	–
*Lactobacillus*	0.004	0.308	0.008	0.009/0.223	0.007/0.109	–	0.003/0.009
*Synergistes*	0.002	0.052	0.018	0.010/0.223	0.006/0.009	0.004/0.009	–
*Lachnospira*^a^	5.049	2.952	2.297	0.022/0.401	0.031/0.046	0.008/0.024	–
*Prevotella*^a^	10.289	5.296	15.251	0.028/0.404	0.079/0.120	–	0.009/0.027
*Succinivibrio*	0.353	0.000	0.118	0.030/0.404	0.029/0.085	0.082/0.124	–
**Metabolites**				***P*/FDR _(N vs FL1 vs FL2)_**	***P*/FDR _(N vs FL1)_**	***P*/FDR _(N vs FL2)_**	***P*/FDR _(FL1 vs FL2)_**
UDCA	105.41	167.42	209.89	0.08/0.85	0.05/0.85	0.04/0.85	–

N, healthy control group; FL1, mild fatty liver; FL2, moderate-to-severe fatty liver; FDR, false discovery rate; UDCA, ursodeoxycholic acid.

**Note:** Bacterial taxa are reported as relative abundance, and fecal metabolites as normalized levels. Values in the comparison columns are shown as raw *P* value/ Benjamini–Hochberg false discovery rate (BH-FDR)–adjusted q value. The metabolite result is exploratory because it did not remain significant after BH-FDR correction. ∗∗∗raw *P* < 0.001.

a Indicates one of the ten most abundant bacterial taxa; — indicates raw *P* > 0.1.

In contrast, *Prevotella* abundance tended to be higher in the FL2 group than in the FL1 group, whereas *Lactobacillus* showed a higher abundance in FL1 but a lower abundance in FL2. Among bile acid–related metabolites, ursodeoxycholic acid (UDCA) showed only nominal differences across CAP-defined steatosis groups, and this finding did not remain significant after BH-FDR correction. Given the modest size of the FL1 and FL2 groups, the absence of a clear severity-related microbiota pattern should be interpreted cautiously and should not be taken as evidence that microbiota changes are independent of steatosis severity.

### Diabetes-associated Gut Microbiota and Metabolite Dysbiosis in NAFLD Patients

Type 2 diabetes was defined according to established diagnostic criteria, including a fasting plasma glucose level ≥7.0 mmol/L (126 mg/dL). In the present cohort, all 27 NAFLD patients classified as having type 2 diabetes were newly diagnosed at enrollment and were treatment-naïve; none had a prior history of antidiabetic therapy, including metformin or other glucose-lowering medications.

At the phylum level, Synergistetes abundance was increased in NAFLD patients, with a more pronounced increase observed in those with diabetes (Figure 3). Actinobacteria abundance was also higher in diabetic patients compared with non-diabetic NAFLD patients. At the genus level, 12 genera showed nominal differences among groups, with selected comparisons remaining significant after FDR correction (Table 3). Consistent with patterns reported in other metabolic disorders, butyrate-producing genera such as *Lachnospira* and *Roseburia* were lower in patients with diabetes, whereas *Megamonas* was higher (Figure 4). Several taxa, including *Cloacibacillus* and *Megasphaera*, showed nominal differences according to diabetes status, with higher relative abundance in FL-DM than in FL-NDM before FDR correction.

**Table 3:** Differentially Abundant Bacterial Taxa and Exploratory Metabolite Differences in Healthy Controls and NAFLD Patients Stratified by Diabetes Status.

	N	FL-NDM	FL-DM	*P*/FDR ^(N vs FL−DM vs FL−NDM)^	*P*/FDR _(N vs FL-DM)_	*P*/FDR _(N vs FL-NDM)_	*P*/FDR ^(FL−DM vs FL−NDM)^
n	26	39	27				
Phylum							
Synergistetes^a^	0.004	0.017	0.083	0.002/0.023	∗∗∗/0.010	0.018/0.197	0.059/0.356
Actinobacteria^a^	2.115	0.965	3.248	–	–	–	0.036/0.355
Genus							
*Lachnospira*^a^	5.023	3.517	1.259	0.002/0.056	∗∗∗/0.019	–	0.017/0.195
*Roseburia*^a^	5.700	6.074	1.984	0.002/0.062	∗∗∗/0.019	–	0.004/0.187
*Cloacibacillus*	0.0003	0.001	0.015	0.005/0.116	∗∗∗/0.062	–	0.038/0.058
*Synergistes*	0.002	0.012	0.065	0.008/0.131	∗∗∗/0.075	0.006/0.322	–
*Megamonas*^a^	5.258	8.238	9.945	0.009/0.131	0.079/0.627	–	0.003/0.187
*Faecalibacterium*^a^	13.897	6.719	7.022	0.010/0.139	0.015/0.191	0.005/0.322	–
*Megasphaera*	0.594	1.531	6.346	0.012/0.149	0.011/0.158	–	0.009/0.189
*Oxalobacter*	0.01	0.002	0.010	0.024/0.263	–	–	0.008/0.189
*Succinivibrio*	0.355	0.109	0.000	0.030/0.267	0.038/0.423	0.065/0.696	–
*Odoribacter*	0.043	0.018	0.054	0.033/0.267	–	–	0.011/0.188
*Anaerostipes*	0.221	0.140	0.041	∗∗∗/0.026	∗∗∗/0.008	0.018/0.489	0.024/0.247
*Pseudoramibacter_Eubacterium*	0.0003	0.002	0.010	0.001/0.034	∗∗∗/0.019	0.044/0.697	0.015/0.189
**Metabolites**				***P*/FDR ^(N vs FL−DM vs FL−NDM)^**	***P*/FDR _(N vs FL-DM)_**	***P*/FDR _(N vs FL-NDM)_**	***P*/FDR ^(FL−DM vs FL−NDM)^**

Butyrate	429756.36	428016.95	423327.12	∗∗∗/0.08	∗∗∗/0.08	–	∗∗∗/0.08
Caproate	2679.19	2733.35	4830.91	0.01/0.11	∗∗∗/0.08	–	0.01/0.11
Isobutyrate	173849.7	171785.68	167973.52	∗∗∗/0.09	∗∗∗/0.08	–	0.01/0.11
Isovalerate	8268.76	7336.24	10855.28	∗∗∗/0.13	–	–	∗∗∗/0.08
Valerate	4068.82	3613.58	5421.68	∗∗∗/0.13	0.05/0.26	–	∗∗∗/0.08
TCA	5.71	11.51	4.97	∗∗∗/0.08	–	0.02/0.12	∗∗∗/0.08
TCDCA	12.9	13.57	8.19	0.03/0.15	0.02/0.13	–	0.02/0.13
UDCA	105.41	169.57	225.92	0.05/0.22	0.03/0.17	–	–
l-Car	5.01	4.45	4.45	0.03/0.15	–	0.02/0.13	0.04/0.17

N, healthy control group; FL-NDM, fatty liver without DM; FL-DM, fatty liver with diabetes; FDR, false discovery rate; TCA, taurocholic acid; TCDCA, taurochenodeoxycholic acid; UDCA, ursodeoxycholic acid; L-Car, l-carnitine.

**Note:** Bacterial taxa are reported as relative abundance, and fecal metabolites as normalized levels. Values in the comparison columns are shown as raw *P* value/ Benjamini–Hochberg false discovery rate (BH-FDR)–adjusted q value. Metabolite differences are reported as nominal and exploratory because none remained significant after BH-FDR correction. ∗∗∗raw *P* < 0.001.

a Indicates one of the ten most abundant bacterial taxa; — indicates raw *P* > 0.1.

Exploratory metabolite analysis showed nominal differences in nine metabolites between groups; however, none remained significant after BH-FDR correction (Table 3). Before FDR correction, nominal differences were observed in butyrate-related metabolites, including butyrate, isobutyrate, caproate, and valerate, and in bile acid–related metabolites, including taurochenodeoxycholic acid (TCDCA) and UDCA, mainly in relation to diabetes status. L-Carnitine and taurocholic acid (TCA) also showed nominal patterns across groups, but these findings were not statistically significant after BH-FDR correction.

Overall, the bacterial findings, together with exploratory metabolite patterns, place the butyrate-related changes within a broader metabolic-disease context. In this cohort, the main observation was not the reduction of butyrate-producing bacteria itself, but that this pattern was more evident in NAFLD patients with diabetes than in those with overweight alone.

### Gut Microbiota and Metabolite Alterations Associated with Overweight in NAFLD (BMI >25 kg/m^2^)

At the phylum level, Synergistetes abundance differed across healthy controls, non-overweight NAFLD patients, and overweight NAFLD patients, with the highest abundance observed in the non-overweight NAFLD subgroup. Actinobacteria abundance was lower in overweight NAFLD patients (Figure 3; Table 4). At the genus level, *Blautia* and *Roseburia* showed lower abundance in overweight individuals. Several genera, including *Anaerostipes*, *Synergistes*, *Faecalibacterium*, *Lachnospira*, *Pseudoramibacter_Eubacterium*, *Cloacibacillus*, *Dialister*, *Succinivibrio*, and *Bifidobacterium*, differed across healthy controls, non-overweight NAFLD patients, and overweight NAFLD patients (Figure 4; Table 4). Among metabolites, UDCA showed nominal differences across fatty liver and overweight subgroups, and isobutyrate showed a nominal decrease in the overweight subgroup compared with healthy controls. These metabolite findings were exploratory because they did not remain significant after BH-FDR correction.

**Table 4:** Differentially Abundant Bacterial Taxa and Exploratory Metabolite Differences According to Overweight Status in NAFLD.

	N	FL-NOB	FL-OB	*P*/FDR _(N vs FL-NOB vs FL-OB)_	*P*/FDR _(N vs FL-NOB)_	*P*/FDR _(N vs FL-OB)_	*P*/FDR _(FL-NOB vs FL-OB)_
n	26	28	38				
Phylum							
Synergistetes^a^	0.004	0.073	0.023	0.009/0.058	0.006/0.068	0.005/0.058	–
Actinobacteria^a^	2.115	3.370	0.834	0.010/0.058	0.073/0.402	–	0.002/0.027
Genus							
*Anaerostipes*	0.221	0.127	0.080	0.002/0.071	0.011/0.196	∗∗∗/0.057	–
*Pseudoramibacter_Eubacterium*	0.000	0.010	0.002	0.002/0.071	∗∗∗/0.067	0.038/0.473	0.048/0.501
*Blautia*	3.275	2.212	1.031	0.002/0.071	–	0.024/0.338	∗∗∗/0.085
*Synergistes*	0.002	0.058	0.016	0.004/0.108	0.001/0.076	0.014/0.282	–
*Faecalibacterium*^a^	13.958	6.956	6.825	0.011/0.227	0.009/0.183	0.008/0.283	–
*Dialister*	0.687	1.912	1.702	0.015/0.236	0.005/0.173	–	0.038/0.501
*Eggerthella*	0.009	0.009	0.015	0.015/0.236	–	–	0.004/0.214
*Cloacibacillus*	0.000	0.014	0.002	0.021/0.284	0.008/0.183	–	–
*Lachnospira*^a^	5.038	2.692	2.534	0.025/0.293	0.022/0.300	0.014/0.283	–
*Bifidobacterium*	1.903	2.681	0.670	0.028/0.293	–	–	0.007/0.240
*Succinivibrio*	0.353	0.000	0.112	0.030/0.293	0.034/0.361	–	–
*Christensenella*	0.001	0.008	0.002	0.034/0.302	–	–	0.009/0.240
*Roseburia*^a^	5.664	6.337	2.972	0.049/0.381	–	0.014/0.283	–
*Haemophilus*	0.099	0.008	0.100	0.049/0.381	–	–	0.012/0.248
**Metabolites**				***P*/FDR _(N vs FL-NOB vs FL-OB)_**	***P*/FDR _(N vs FL-NOB)_**	***P*/FDR _(N vs FL-OB)_**	***P*/FDR _(FL-NOB vs FL-OB)_**

UDCA	105.41	240.19	165.28	0.01/0.66	∗∗∗/0.56	0.02/0.75	0.05/0.74
Isobutyrate	173849.7	168182.53	167973.86	–	–	0.04/0.74	–

NAFLD, non-alcoholic fatty liver disease; N, healthy control group; N, healthy control group; FL-NOB, fatty liver without overweight; FL-OB, fatty liver with overweight; FDR, false discovery rate; UDCA, ursodeoxycholic acid. Overweight was defined as body mass index (BMI) >25 kg/m^2^.

**Note:** Bacterial taxa are reported as relative abundance, and fecal metabolites as normalized levels. Values in the comparison columns are shown as raw *P* value/Benjamini–Hochberg false discovery rate (BH-FDR)–adjusted q value. Metabolite differences are reported as nominal and exploratory because none remained significant after BH-FDR correction. ∗∗∗raw *P* < 0.001.

a Indicates one of the ten most abundant bacterial taxa; — indicates raw *P* > 0.1.

### Exploratory Analysis of Combined Overweight and Diabetes Status

Because some subgroups were small, especially the fatty liver with non-overweight and diabetes subgroup (FL-NOB-DM, n = 11), the combined overweight–diabetes stratification was considered exploratory. The detailed bacterial and metabolite comparisons across the five subgroups are provided in Supplementary Table S2. Overall, the combined stratification showed patterns broadly consistent with the main diabetes- and overweight-based analyses, with diabetes-related subgroups showing more evident changes in butyrate-producing genera and exploratory nominal patterns in related metabolites. However, these subgroup findings should be interpreted cautiously because of limited statistical power.

### Correlations Between Gut Dysbiotic Bacteria, Metabolites, and Metabolic Traits

**Figure 5:**
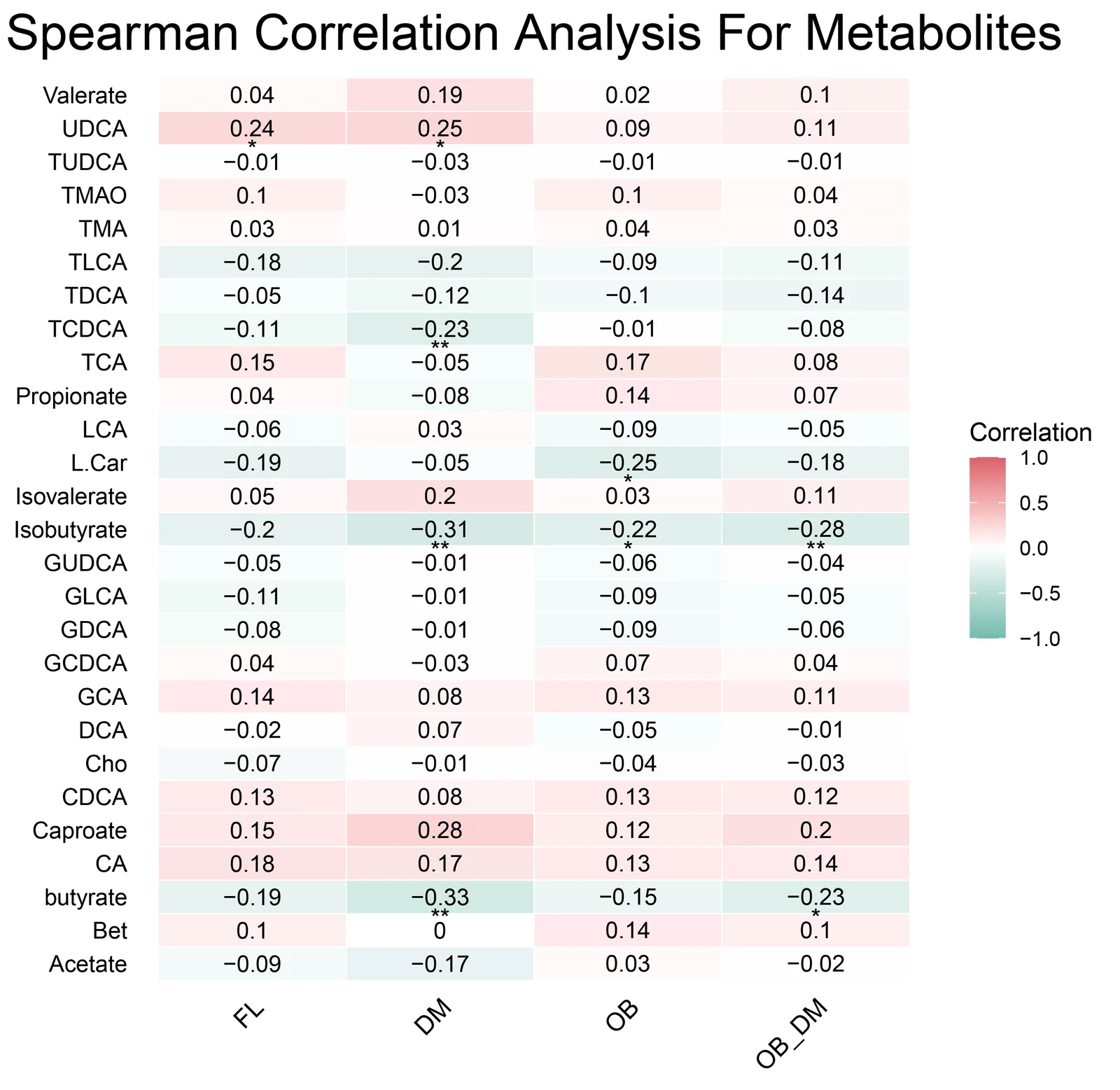
**Spearman correlation analysis between fecal metabolites and NAFLD-related metabolic status variables**. **Note:** Spearman correlation heatmap showing exploratory associations between fecal metabolites and NAFLD-related metabolic status variables. Columns represent NAFLD status (FL), diabetes status (DM), overweight status (OB), and combined overweight–diabetes status (OB-DM). Rows represent quantified fecal metabolites, including short-chain fatty acids, bile acids, and choline-related metabolites. Numbers in each cell indicate Spearman correlation coefficients. Positive correlations are shown in red and negative correlations in green. Asterisks indicate nominal statistical significance before multiple-testing correction: ∗*P* < 0.05 and ∗∗*P* < 0.01. These correlations were exploratory and were not adjusted using multivariable regression models. NAFLD: non-alcoholic fatty liver disease; FL: fatty liver.

**Figure 6:**
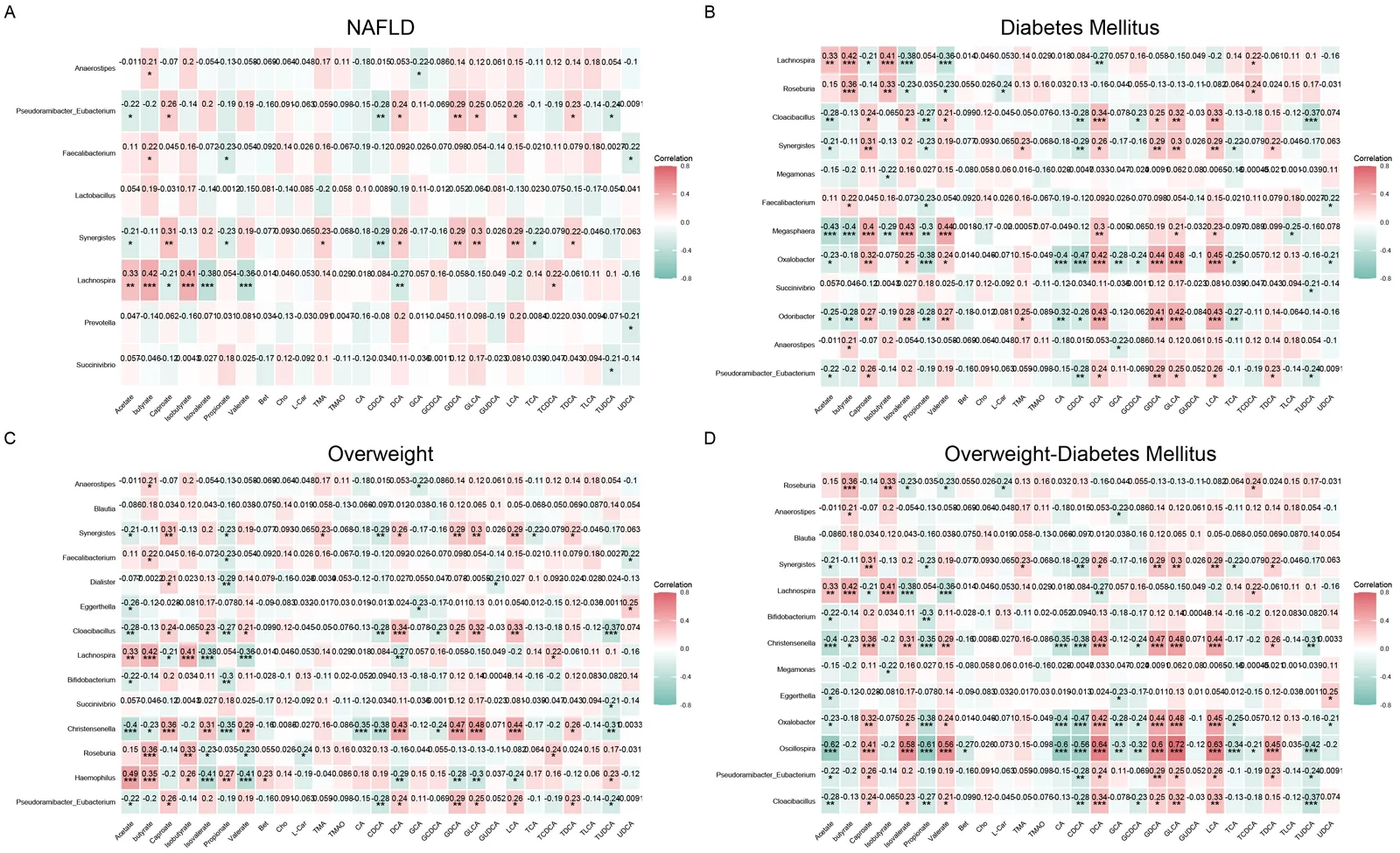
**Correlation analysis between dysbiosis-associated bacterial taxa and fecal metabolites.** A, NAFLD; B, DM; C, OB; D, OB-DM. Correlations were assessed using Spearman correlation analysis. NAFLD: non-alcoholic fatty liver disease; DM: diabetes; OB: overweight; OB-DM: overweight with diabetes.

SCFAs, bile acids, and choline-related metabolites were quantified, and their associations with metabolic status and gut microbiota were evaluated using Spearman correlation analysis (Figure 5, Figure 6). Overall, diabetes and overweight showed distinct but overlapping correlation patterns with both metabolites and bacterial taxa.

In exploratory Spearman correlation analysis, butyrate and isobutyrate were negatively correlated with diabetes, and isobutyrate also showed a negative association with overweight. In the presence of overweight, the correlation between isobutyrate and metabolic status was stronger than that of butyrate. Lower butyrate levels were observed together with lower abundance of known butyrate-producing genera, including *Roseburia*, *Lachnospira*, *Faecalibacterium*, and *Anaerostipes*, whereas taxa such as *Megamonas*, *Megasphaera*, and *Odoribacter* showed inverse associations in diabetic patients.

Valerate-related metabolites showed nominally higher levels in diabetes and in combined diabetes with overweight. In exploratory correlation analysis, these metabolites were positively associated with genera such as *Cloacibacillus*, *Megasphaera,* and *Odoribacter*, while *Roseburia* and *Lachnospira* showed negative correlations.

Bile acid–related metabolites showed exploratory correlation patterns. Several genera, including *Synergistes*, *Pseudoramibacter_Eubacterium*, *Lachnospira*, and *Cloacibacillus*, were consistently associated with bile acid–related metabolites in both diabetes and overweight. In exploratory correlation analysis, TCA appeared more related to overweight status, whereas TCDCA appeared more related to diabetes.

In contrast, choline-related metabolites showed limited changes, although L-carnitine levels were reduced in overweight individuals and exhibited correlations with specific gut bacteria.

Overall, correlation analyses showed links between selected bacterial genera and metabolite profiles, particularly involving butyrate- and bile acid–related metabolites. These exploratory findings suggest possible microbiota–metabolite patterns related to diabetes and overweight in NAFLD, but they require validation in larger cohorts.

### Associations Between Gut Microbiota, Metabolites, and Clinical Metabolic Indicators

**Figure 7:**
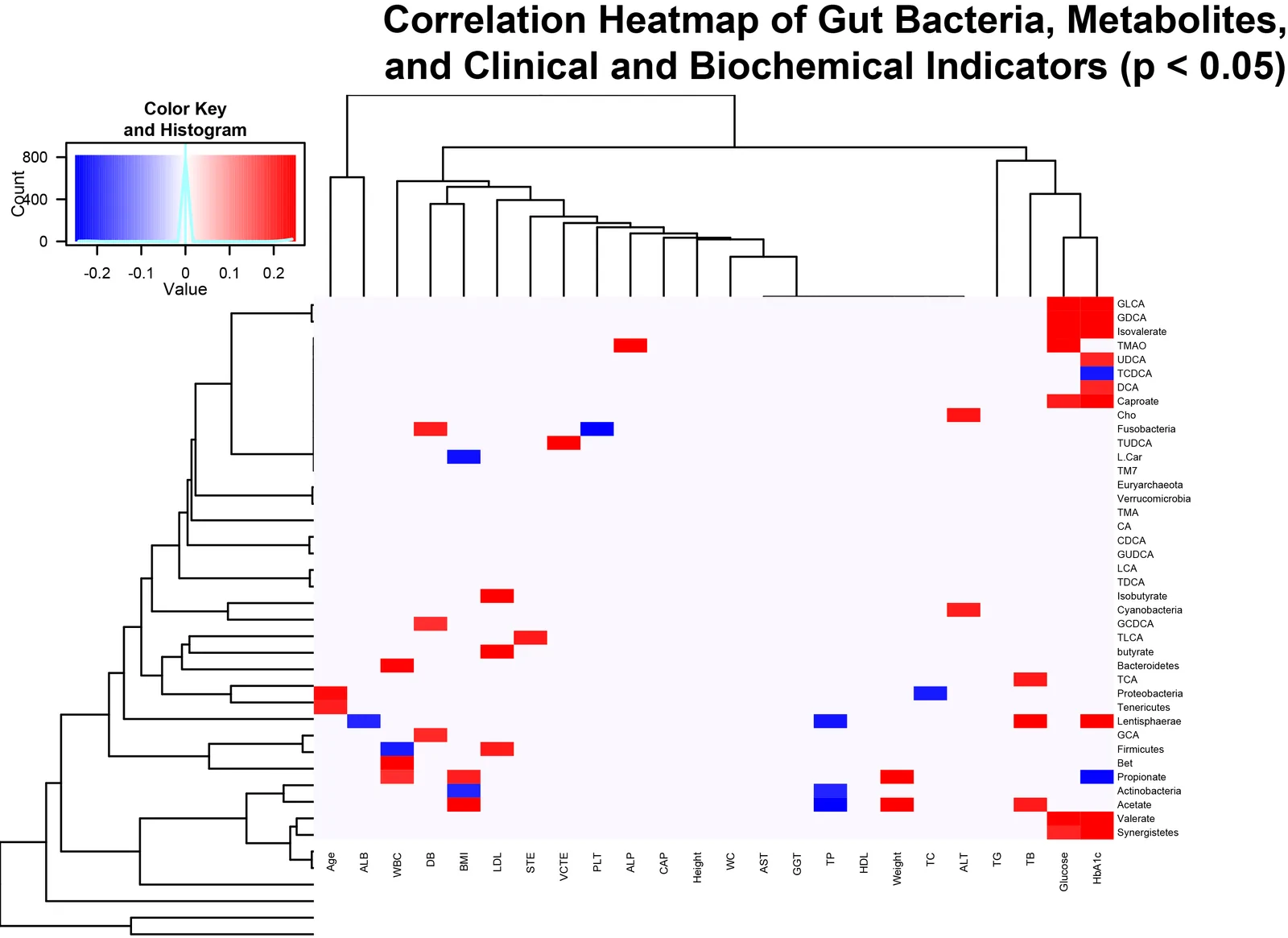
**Correlation heatmap of gut microbiota, fecal metabolites, and clinical and biochemical indices**. Correlations were assessed using Spearman correlation analysis. Each row represents a microbial taxon or fecal metabolite, and each column represents a clinical, biochemical, or elastography-related indicator. Only nominally significant correlations are shown.

As shown in Figure 7, Spearman correlation analysis showed associations between selected gut microbial taxa, fecal metabolites, and clinical indicators, including glucose, HbA1c, BMI, LDL, and liver stiffness. Several microbial taxa and fecal metabolites showed parallel exploratory correlations with glucose and HbA1c. Liver stiffness also showed correlations with several fecal metabolites. These results suggest exploratory associations between gut microbiota, fecal metabolites, and key metabolic indicators.

### Exploratory Discrimination Performance of Gut Microbiota and Metabolite Signatures

**Figure 8:**
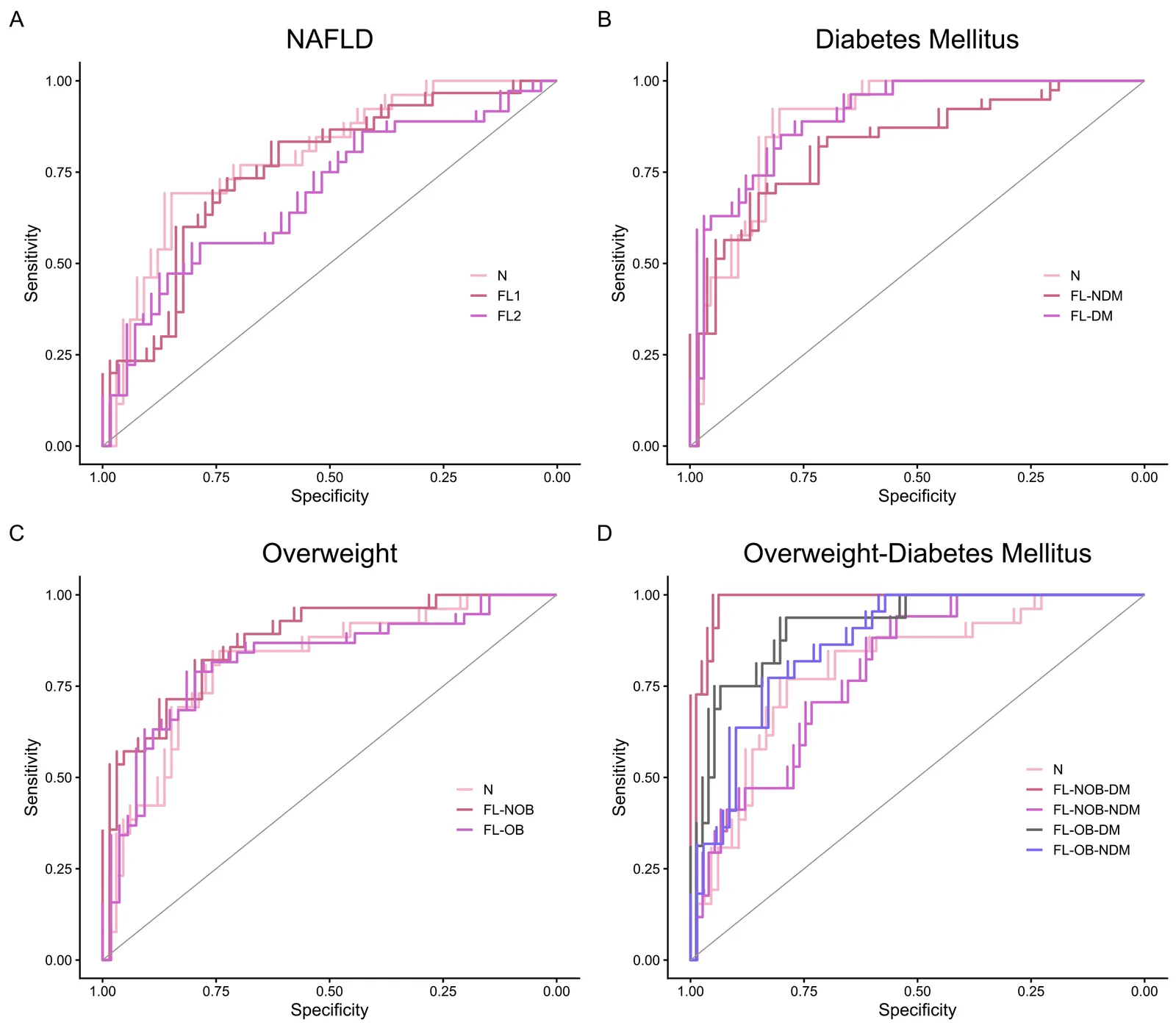
**Exploratory discrimination models based on gut bacterial taxa and fecal metabolites**. A, NAFLD; B, DM; C, OB; D, OB-DM. The models were exploratory and were not internally or externally validated; therefore, the ROC/AUC values reflect apparent performance and should not be interpreted as validated diagnostic tools. NAFLD: Non-alcoholic fatty liver disease; DM: diabetes; OB: overweight; OB-DM: overweight with diabetes; ROC: receiver operating characteristic curve; AUC: area under the receiver operating characteristic curve; N: healthy control group; FL1: mild fatty liver; FL2: moderate-to-severe fatty liver; FL-DM: fatty liver with diabetes; FL-NDM: fatty liver without diabetes; FL-OB: fatty liver with overweight; FL-NOB: fatty liver without overweight; FL-OB-DM: fatty liver with overweight with diabetes; FL-OB-NDM: fatty liver with overweight without diabetes; FL-NOB-DM: fatty liver with non-overweight with diabetes; FL-NOB-NDM: fatty liver with non-overweight without diabetes.

Using random forest analysis, we ranked bacterial features by importance and selected the top 30 features for exploratory model construction. Together with metabolites showing nominal differences, these bacterial features were used to build exploratory discrimination models for NAFLD, diabetes, overweight, and combined overweight–diabetes status. Apparent model performance in the study cohort is summarized in Table 5 and Figure 8.

**Table 5:** Apparent Exploratory ROC Performance of Gut Bacterial Taxa and Fecal Metabolites for Discriminating NAFLD, Diabetes, Overweight, and Combined Overweight–Diabetes Status.

		AUC	AUC 95% CI	Specificity (%)	Sensitivity (%)	Accuracy (%)
NAFLD	N	0.80	0.70–0.90	86.36	69.23	81.52
	FL1	0.77	0.67–0.87	62.90	83.33	69.57
	FL2	0.70	0.59–0.82	80.36	55.56	70.65
DM	N	0.90	0.84–0.96	81.82	92.31	84.78
	FL-DM	0.91	0.85–0.97	81.54	85.19	82.61
	FL-NDM	0.84	0.76–0.92	71.70	84.62	77.17
OB	N	0.84	0.75–0.92	72.73	84.62	76.09
	FL-NOB	0.88	0.81–0.96	93.75	67.86	85.87
	FL-OB	0.86	0.78–0.94	85.19	81.58	83.70
OB-DM	N	0.81	0.71–0.91	80.30	76.92	79.35
	FL-NOB-DM	0.99	0.98–1.00	95.06	100.00	95.65
	FL-NOB-NDM	0.80	0.70–0.91	56.00	94.12	63.04
	FL-OB-DM	0.92	0.86–0.99	80.26	93.75	82.61
	FL-OB-NDM	0.88	0.81–0.95	84.29	77.27	82.61

ROC, receiver operating characteristic curve; NAFLD, non-alcoholic fatty liver disease; AUC, area under the receiver operating characteristic curve; CI, confidence interval; N, healthy control group; FL1, Mild fatty liver; FL2, moderate-to-severe fatty liver; DM, diabetes mellitus; FL-DM, fatty liver with diabetes; FL-NDM, fatty liver without diabetes; OB, overweight; FL-NOB, fatty liver without overweight; FL-OB, fatty liver with overweight; OB-DM, overweight with diabetes; FL-NOB-DM, fatty liver without overweight and with diabetes; FL-NOB-NDM, fatty liver without overweight and no diabetes; FL-OB-DM, fatty liver with overweight with diabetes; FL-OB-NDM, fatty liver with overweight and without diabetes.

**Note:** These exploratory models were fitted in the study cohort, and no internal or external validation was performed. The reported AUC values therefore reflect apparent performance and may be optimistic. The very high AUC observed in the FL-NOB-DM subgroup should be interpreted with particular caution because this subgroup included only 11 participants and the model was based on 30 selected features, making it prone to overfitting. These models should not be interpreted as validated diagnostic tools.

These results suggest that selected microbial and metabolite features showed apparent discrimination ability within the study cohort. However, the very high performance observed in the FL-NOB-DM subgroup should be interpreted with particular caution, because this subgroup included only 11 participants and the model was based on 30 selected features. Such results are likely to be unstable and may reflect overfitting rather than robust diagnostic performance. Because no internal or external validation was performed, the models should not be interpreted as validated diagnostic tools.

## Discussion

In this study, we examined gut microbiota and fecal metabolite profiles in patients with imaging-defined NAFLD under different metabolic conditions. Overall, diabetes was associated with more evident gut microbiota differences than overweight alone, while metabolite findings should be viewed as exploratory. This supports the view that metabolic comorbidities contribute to heterogeneity in NAFLD-related microbiota profiles.^15^ Previous studies have suggested that NAFLD-related gut microbiota and bile acid profiles may vary across metabolic phenotypes. Chen *et al.* also reported that lean NAFLD differed from non-lean NAFLD in bile acid–related metabolic adaptation and gut microbiota profiles.^16^ More recently, a South Asian study comparing lean and non-lean/obese NAFLD also reported distinct gut microbial signatures in lean NAFLD, further supporting the concept that NAFLD-related microbiota patterns vary according to metabolic phenotype.^17^ In this context, our study focused on overweight and diabetes status, supporting the idea that microbiota–metabolite patterns in NAFLD should be interpreted according to metabolic background rather than as a uniform disease signature.

We also compared CAP-defined mild and moderate-to-severe fatty liver groups. Although some taxa differed between healthy controls and NAFLD patients, no consistent microbiota gradient was observed between FL1 and FL2. This finding should be interpreted cautiously, as FL1/FL2 grouping was based on CAP-defined steatosis rather than histological staging, and the subgroup sizes were modest.

Our exploratory metabolite analysis suggested nominal differences in SCFAs and related metabolites across metabolic subgroups.^18^ Butyrate and isobutyrate tended to be lower in patients with diabetes and in those with combined overweight and diabetes, while caproate and valerate showed different patterns in diabetic patients.^19^^,^^20^ Because these metabolite differences did not remain significant after BH-FDR correction, they should not be interpreted as confirmatory metabolic alterations.

Reductions in butyrate-producing bacteria have been reported in obesity, diabetes, NAFLD, and other metabolic disorders^15^^,^^21^, ^22^, ^23^, ^24^. Therefore, we do not regard this finding as a disease-specific microbial marker for NAFLD with diabetes. The value of the present analysis is mainly comparative: this known butyrate-related dysbiosis pattern appeared more evident in NAFLD patients with diabetes than in those with overweight alone. In our cohort, lower butyrate levels were observed alongside lower abundance of *Anaerostipes*, *Faecalibacterium*, *Lachnospira*, and *Roseburia*, together with higher abundance of *Megasphaera*, *Odoribacter*, and *Megamonas*.^21^^,^^25^ These findings are consistent with previous reports but remain associative, as intestinal barrier integrity, host inflammatory pathways, and histological liver injury were not assessed.

Several bacterial taxa previously reported in metabolic or inflammatory conditions also showed different abundance patterns in diabetic NAFLD patients. *Megasphaera*, *Odoribacter*, and *Megamonas* have been described in metabolic disorders and inflammatory conditions.^23^^,^^24^^,^^26^ In this study, their higher abundance in diabetic patients suggests an association with diabetic metabolic status. Bile acid– and amino acid–related metabolites also showed exploratory differences, consistent with previous reports linking gut microbiota–derived metabolites with NAFLD and diabetes.^27^^,^^28^ However, without functional metagenomics, metatranscriptomics, host inflammatory markers, or liver histology, these findings should be considered hypothesis-generating rather than mechanistic evidence.

This study was conducted in a complex clinical setting. Although major microbiome-related confounders, including recent medication use, recent weight change, and significant alcohol intake, were excluded at enrollment, long-term dietary habits and physical activity were not quantitatively assessed. Residual lifestyle-related confounding may therefore remain. From a clinical perspective, our findings suggest that metabolic context should be considered when interpreting NAFLD-associated microbiota–metabolite profiles. However, these exploratory cross-sectional data are not sufficient to guide microbiome-based treatment, and larger longitudinal or interventional studies are needed before personalized strategies can be considered.

### Limitations

This study has several limitations. First, its observational design prevents causal inference. Because BMI cutoffs for overweight may vary by ethnicity and regional guidelines, the overweight-related findings should be interpreted according to the BMI >25 kg/m^2^ definition used in this study. The use of Asian-specific BMI thresholds may alter subgroup allocation and should be considered in future validation studies. Liver biopsy was not performed, so we could not distinguish simple steatosis from steatohepatitis or stage fibrosis histologically. Although CAP, FibroScan-based liver stiffness measurement, and shear wave elastography were used to assess steatosis and exclude participants with evidence of significant fibrosis or cirrhosis, these methods cannot fully replace histological evaluation. Therefore, our findings should be interpreted in the context of imaging-defined NAFLD and CAP-defined steatosis categories, rather than biopsy-confirmed NASH, inflammatory activity, or fibrosis stage. Similarly, the FL1/FL2 comparison was limited by sample size and CAP-based grouping, so the lack of a clear microbiota gradient should not be interpreted as true severity independence.

Second, although several microbiome-related confounders were excluded at enrollment, including recent use of antibiotics, probiotics/prebiotics, metformin or other antidiabetic medications, and significant alcohol intake, long-term dietary composition and physical activity were not quantitatively assessed. Residual lifestyle-related confounding may therefore remain, particularly in subgroup comparisons. In addition, because of the exploratory design and limited subgroup sample sizes, multivariable regression models adjusting for age, sex, BMI, HbA1c, triglycerides, HDL, LDL, WC, or waist-to-hip ratio were not performed. Therefore, the observed diabetes-associated microbiota patterns may partly reflect differences in obesity, glycemic status, dyslipidemia, abdominal adiposity, age, sex, or other metabolic features, rather than the independent effect of diabetes itself. These findings should be interpreted as associations observed within a complex metabolic background, and larger studies are needed to determine whether these microbial patterns remain independently associated with diabetes after adjustment for key clinical covariates.

Third, the sample size was relatively small, especially after combined stratification by overweight and diabetes status, where the smallest subgroup was FL-NOB-DM (n = 11). These subgroup analyses were underpowered and should be regarded as exploratory. In addition, microbiota profiling was based on 97% OTU clustering rather than Amplicon Sequence Vaariant–based methods. Multivariable regression and compositional approaches such as ALDEx2 (ANOVA-Like Differential Expression tool for high-throughput sequencing) or (Analysis of Composition of Microbiomes) were not applied, and Spearman correlation analyses may not fully account for confounding or the compositional nature of microbiome data. Although alpha diversity rarefaction curves were generated and relative abundance normalization was used, the original platform report did not provide a fixed rarefaction depth. Finally, the discrimination models lacked both internal validation, such as k-fold cross-validation, bootstrap validation, or repeated random splitting, and external validation. The very high AUC observed for the FL-NOB-DM subgroup should be interpreted with particular caution because this subgroup included only 11 participants, whereas the exploratory model used 30 selected features. In this setting, the apparent AUC and sensitivity may be unstable, exaggerated, and prone to overfitting. Therefore, the reported ROC/AUC values should be considered exploratory and hypothesis-generating rather than evidence of clinically useful diagnostic performance. Larger independent cohorts are needed to validate these models.

This exploratory study suggests that gut microbiota and fecal metabolite profiles in NAFLD may differ according to metabolic comorbidities, with T2D showing a stronger association than overweight alone. These findings highlight the need to consider metabolic heterogeneity, particularly glycemic status, when interpreting gut microbiota–metabolite signatures in imaging-defined NAFLD. The discrimination models should be regarded as hypothesis-generating only, especially in small subgroups, and require validation in larger independent cohorts before any clinical application can be considered.

## Data availability

The sequencing data supporting the findings of this study have been deposited in the China National GeneBank DataBase (CNGBdb) under project accession number CNP0007571.

The gut microbiota sequencing data are available under subproject sub073005, and the metabolomics data are available under subproject sub073040.

All data are publicly accessible through the CNGBdb repository at: https://db.cngb.org/search/project/CNP0007571/

## Funding

This work was by the Clinical Scientist Training Program of Shenzhen People’s Hospital (Grant number SYWGSCGZH202202) and Shenzhen International Cooperation Research Project (Approval No. GJHZ20240218114459012).

## CRediT authorship contribution statement

Jieying Zeng: Conceptualization, Methodology, Investigation, Data curation, Formal analysis, Writing – original draft. Keen Yang: Investigation, Data curation, Formal analysis, Writing – original draft. Huaiyu Wu: Investigation, Data curation. Ziwei Lin: Investigation, Data curation. Lisi Liao: Investigation, Data curation. Fajin Dong: Conceptualization, Methodology, Supervision, Writing – review & editing. Yaxin Zhu: Methodology, Supervision, Writing – review & editing.

## Declaration of competing interest

The authors declare the following financial interests/personal relationships, which may be considered as potential competing interests: Jieying Zeng reports article publishing charges were provided by the Shenzhen International Cooperation Research Project. If there are other authors, they declare that they have no known competing financial interests or personal relationships that could have appeared to influence the work reported in this paper.
