# Supplementary material for: Diabetes-associated Alterations in the Gut Microbiota–metabolite Profile of NAFLD Under Complex Clinical Conditions
Source: J Clin Exp Hepatol. 2026 Aug 19;16(6):103637. doi: 10.1016/j.jceh.2026.103637 (PMC13583973; doi:10.1016/j.jceh.2026.103637)

Figure S1:Flowchart of participant recruitment, exclusion, and final inclusion in the study

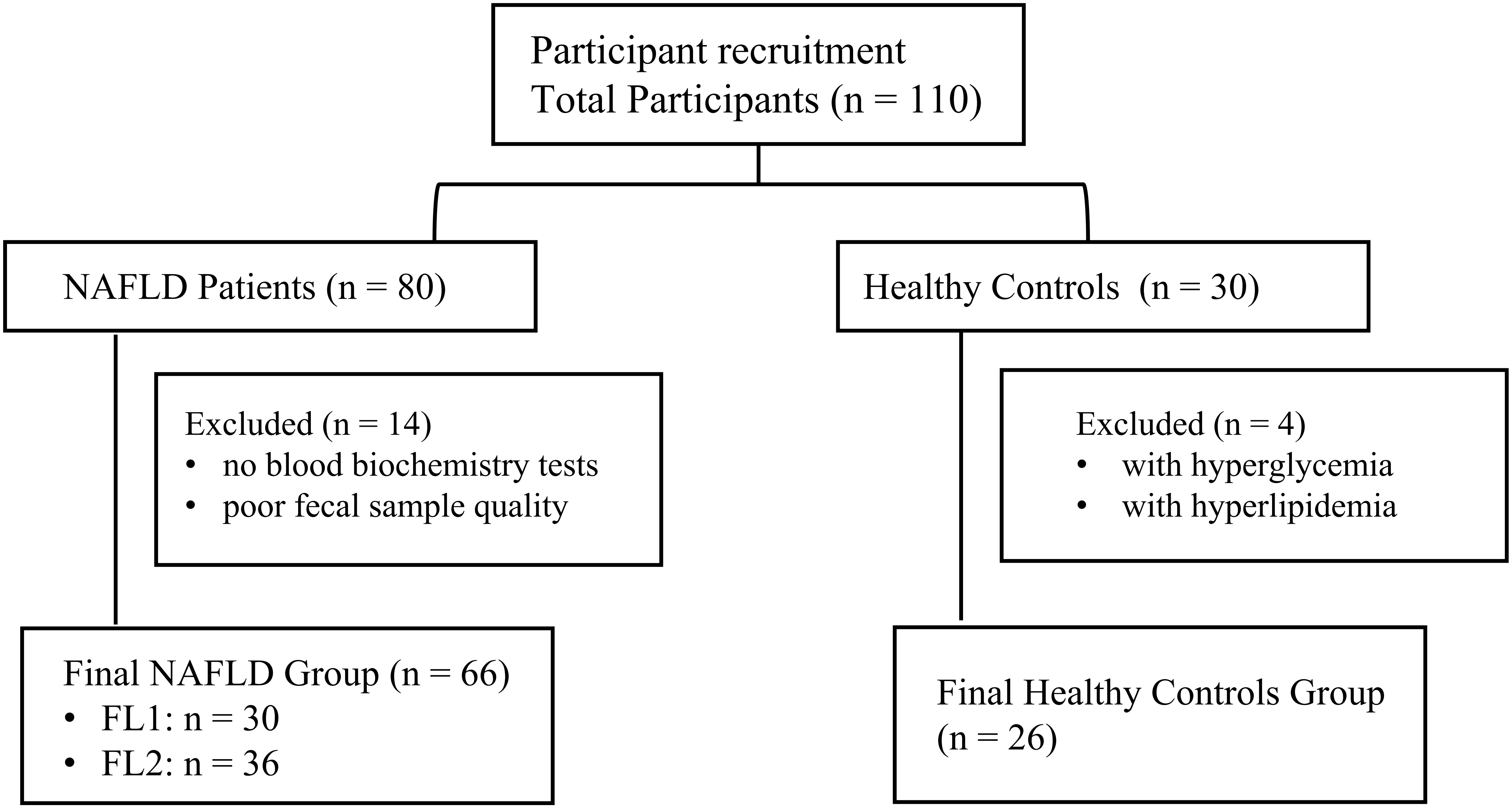

Supplement: Supplementary file 1 [file mmc3.pdf]
