# Supplementary material for: Diabetes-associated Alterations in the Gut Microbiota–metabolite Profile of NAFLD Under Complex Clinical Conditions
Source: J Clin Exp Hepatol. 2026 Aug 19;16(6):103637. doi: 10.1016/j.jceh.2026.103637 (PMC13583973; doi:10.1016/j.jceh.2026.103637)
Supplement: Supplementary file 2 [file mmc1.docx]

**Table S1. Abbreviations Used in the Manuscript**

| **Abbreviation** | **Full term** |
| --- | --- |
| WC | Waist circumference |
| ALB | Albumin |
| ALP | Alkaline phosphatase |
| ALT | Alanine aminotransferase |
| AST | Aspartate aminotransferase |
| BMI | Body mass index |
| CAP | Controlled Attenuation Parameter |
| DB | Direct bilirubin |
| DM | Diabetes mellitus |
| FL | Fatty liver |
| FL1 | Mild fatty liver |
| FL2 | Moderate-to-severe fatty liver |
| FL-DM | Fatty liver with diabetes |
| FL-NDM | Fatty liver without diabetes |
| FL-OB | Fatty liver with overweight |
| FL-NOB | Fatty liver without overweight |
| FL-NOB-DM | Fatty liver with non-overweight and diabetes |
| FL-NOB-NDM | Fatty liver with non-overweight and without diabetes |
| FL-OB-DM | Fatty liver with overweight and diabetes |
| FL-OB-NDM | Fatty liver with overweight and without diabetes |
| GCDCA | Glycochenodeoxycholic acid |
| GGT | γ-glutamyl transpeptidase |
| HbA1c | Glycated hemoglobin |
| HDL | High-density lipoprotein |
| LC–MS/MS | Liquid chromatography–tandem mass spectrometry |
| LDL | Low-density lipoprotein |
| L-Car | L-carnitine |
| MAFLD | Metabolic-associated fatty liver disease |
| NAFLD | Non-alcoholic fatty liver disease |
| N | Healthy control |
| NOB-DM | Non-overweight with diabetes |
| NOB-NDM | Non-overweight without diabetes |
| OB | Overweight |
| OB-DM | Overweight with diabetes |
| OB-NDM | Overweight without diabetes |
| OTU | Operational taxonomic unit |
| PERMANOVA | Permutational multivariate analysis of variance |
| PLT | Platelet |
| ROC | Receiver operating characteristic |
| SCFA | Short-chain fatty acid |
| SD | Standard deviation |
| STE | Sound touch elastography |
| TCA | Taurocholic acid |
| TCDCA | Taurochenodeoxycholic acid |
| TG | Triglyceride |
| TP | Total protein |
| UDCA | Ursodeoxycholic acid |
| WBC | White blood cell |
