# Supplementary material for: Diabetes-associated Alterations in the Gut Microbiota–metabolite Profile of NAFLD Under Complex Clinical Conditions
Source: J Clin Exp Hepatol. 2026 Aug 19;16(6):103637. doi: 10.1016/j.jceh.2026.103637 (PMC13583973; doi:10.1016/j.jceh.2026.103637)
Supplement: Supplementary file 3 [file mmc2.docx]

|  | N | NOB-NDM | NOB-DM | OB-DM | OB-NDM | *P*/FDR (All) | *P*/FDR (N VS NO-ND) | *P*/FDR (N VS NO-DM) | *P*/FDR (N VS OB-DM) | *P*/FDR (N VS OB-ND) | *P*/FDR (NO-ND VS NO-DM) | *P*/FDR (NO-ND VS OB-DM) | *P*/FDR (NO-ND VS OB-ND） | *P*/FDR (NO-DM VS OB-DM) | *P*/FDR (NO-DM VS OB-ND) | *P*/FDR (OB-DM VS OB-ND) |
| --- | --- | --- | --- | --- | --- | --- | --- | --- | --- | --- | --- | --- | --- | --- | --- | --- |
| n | 26 | 17 | 11 | 16 | 22 |  |  |  |  |  |  |  |  |  |  |  |
| Phylum |  |  |  |  |  |  |  |  |  |  |  |  |  |  |  |  |
| Synergistetes^#^ | 0.004 | 0.006 | 0.177 | 0.019 | 0.026 | 0.003/0.032 | —— | ***/0.006 | 0.016/0.193 | 0.011/0.121 | 0.011/0.063 | __ | __ | 0.048/0.205 | 0.049/0.271 | __ |
| Actinobacteria^#^ | 2.118 | 1.658 | 6.015 | 1.385 | 0.432 | 0.006/0.037 | __ | 0.016/0,088 | __ | __ | __ | __ | 0.019/0.214 | 0.050/0.205 | ***/0.003 | __ |
| Cyanobacteria^#^ | 0.002 | 0.000 | 0.003 | 0.001 | 0.008 | 0.025/0.103 | __ | 0.050/0.137 | __ | __ | 0.009/0.063 | __ | __ | 0.006/0.075 | _ | 0.044/0.531 |
| Genus |  |  |  |  |  |  |  |  |  |  |  |  |  |  |  |  |
| *Roseburia^#^* | 2.351 | 8.915 | 2.351 | 1.730 | 3.876 | 0.001/0.036 | __ | 0.007/0.106 | 0.010/0.212 | __ | 0.001/0.145 | 0.002/0.198 | 0.026/0.812 | __ | __ | __ |
| *Anaerostipes* | 0.065 | 0.168 | 0.065 | 0.025 | 0.120 | 0.001/0.036 | __ | 0.005/0.010 | 0.005/0.099 | 0.018/0.675 | 0.057/0.378 | 0.019/0.462 | __ | __ | __ | __ |
| *Blautia* | 3.500 | 1.380 | 3.500 | 1.134 | 0.955 | 0.002/0.039 | __ | 0.025/0.234 | 0.025/0.234 | 0.050/0.835 | 0.011/0.185 | 0.059/0.682 | __ | 0.002/0.115 | 0.001/0.026 | __ |
| *Synergistes* | 0.140 | 0.005 | 0.002 | 0.014 | 0.017 | 0.004/0.070 | 0.022/0.47 | ***/0.015 | ***/0.015 | 0.009/0.674 | 0.035/0.284 | __ | __ | 0.032/0.270 | 0.050/0.335 | __ |
| *Lachnospira* | 0.980 | 3.800 | 0.980 | 1.455 | 3.320 | 0.005/0.070 | __ | 0.002/0.047 | 0.002/0.047 | 0.095/0.834 | 0.007/0.180 | __ | __ | __ | 0.025/0.215 | __ |
| *Gardnerella* | 0.000 | 0.000 | 0.000 | 0.002 | 0.000 | 0.006/0.082 | __ | __ | __ | __ | __ | 0.033/0.639 | __ | __ | __ | 0.016/0.726 |
| *Bifidobacterium* | 5.049 | 1.150 | 5.049 | 1.170 | 0.307 | 0.009/0.095 | __ | 0.023/0.234 | 0.023/0,234 | __ | 0.049/0.375 | __ | 0.049/0.812 | 0.031/0.270 | ***/0.026 | 0.088/0.840 |
| *Christensenella* | 0.016 | 0.003 | 0.016 | 0.002 | 0.001 | 0.009/0.095 | __ | 0.006/0.102 | 0.006/0.102 | __ | 0.018/0.229 | __ | __ | 0.008/0.220 | 0.001/0.026 | __ |
| *Megamonas^#^* | 2.298 | 4.739 | 2.298 | 15.214 | 10.957 | 0.017/0.166 | __ | __ | __ | __ | __ | 0.006/0.309 | __ | 0.041/0.287 | __ | 0.013/0.726 |
| *Eggerthella* | 0.013 | 0.006 | 0.013 | 0.004 | 0.023 | 0.024/0.211 | __ | 0.018/0.218 | 0.018/0.218 | __ | __ | __ | __ | __ | __ | __ |
| *Oxalobacter* | 0.010 | 0.011 | 0.011 | 0.009 | 0.004 | 0.048/0.348 | 0.032/0.47 | __ | __ | __ | 0.007/0.179 | 0.011/0.349 | __ | __ | __ | __ |
| *Oscillospira* | 1.556 | 0.930 | 2.876 | 1.023 | 1.135 | 0.049/0.348 | __ | __ | __ | __ | 0.024/0.234 | __ | __ | 0.017/0.238 | 0.012/0.128 | __ |
| *Pseudoramibacter_Eubacterium* | 0.021 | 0.002 | 0.021 | 0.002 | 0.001 | ***/0.013 | 0.025/0.47 | ***/0.002 | ***/0.002 | __ | 0.012/0.185 | __ | __ | 0.038/0.287 | 0.001/0.026 | __ |
| *Cloacibacillus* | 0.034 | 0.001 | 0.034 | 0.003 | 0.001 | ***/0.013 | __ | ***/0.003 | ***/0.003 | __ | 0.003/0.130 | __ | __ | 0.001/0.220 | 0.004/0.075 | __ |
| Metabolites |  |  |  |  |  |  |  |  |  |  |  |  |  |  |  |  |
| butyrate | 429756.36 | 426238.39 | 414750.34 | 423611.76 | 430803.53 | 0.02/0.10 | __ | 0.02/0.13 | 0.01/0.10 | __ | __ | 0.04/0.15 | __ | __ | 0.03/0.11 | 0.01/0.10 |
| Isobutyrate | 173849.7 | 173452.77 | 167973.86 | 167973.86 | 171521.71 | 0.03/0.12 | __ | __ | ***/0.11 | __ | __ | 0.02/0.10 | __ | 0.01/0.10 | __ | 0.02/0.10 |
| Valerate | 4068.82 | 3628.46 | 5916.18 | 5081.71 | 3602.09 | 0.03/0.54 | __ | __ | __ | __ | __ | 0.04/0.15 | __ | __ | __ | 0.03/0.18 |
| Isovalerate | 8268.76 | 7431.02 | 11838.24 | 10179.5 | 7263 | 0.04/0.25 | __ | __ | __ | __ | __ | 0.04/.16 | __ | __ | __ | 0.03/0.14 |
| TCA | 5.71 | 6.3 | 4.95 | 5.34 | 13.95 | 0.02/0.10 | __ | __ | __ | ***/0.10 | __ | __ | __ | __ | 0.02/0.10 | ***/0.10 |
| TCDCA | 12.9 | 16.75 | 6.25 | 11.35 | 13.46 | 0.02/0.10 | __ | ***/0.13 | 0.04/0.10 | __ | 0.02/0.10 | __ | __ | __ | ***/0.10 | __ |
| GCDCA | 8.5 | 10.59 | 4.37 | 15.79 | 7.91 | 0.04/0.14 | __ | 0.04/0.14 | __ | __ | ***/0.10 | __ | __ | 0.01/0.24 | __ | __ |
| UDCA | 105.41 | 196.57 | 284.09 | 182.08 | 165.28 | 0.04/0.14 | 0.02/0.13 | 0.02/0.13 | __ | __ | __ | __ | __ | __ | 0.03/0.11 | __ |
| L-CA | 5.01 | 4.45 | 4.92 | 4.45 | 4.41 | 0.04/0.14 | __ | __ | __ | 0.01/0.10 | __ | __ | __ | __ | ***/0.10 | __ |

**Supplementary Table S2. Differential bacterial taxa and exploratory fecal metabolite patterns across combined overweight–diabetes subgroups.Note:** NOB-DM, NOB-NDM, OB-DM, and OB-NDM correspond to FL-NOB-DM, FL-NOB-NDM, FL-OB-DM, and FL-OB-NDM in the main text and figures, respectively. Values are shown as mean relative abundance for bacterial taxa and normalized levels for fecal metabolites. Values in comparison columns are shown as raw P value/BH-FDR-adjusted q value. Metabolite findings are exploratory because they did not remain significant after BH-FDR correction. ***raw P < 0.001; # indicates one of the ten most abundant taxa; — indicates raw P > 0.1.
